# Heat Treatment, Impact Properties, and Fracture Behaviour of Ti-6Al-4V Alloy Produced by Powder Compact Extrusion

**DOI:** 10.3390/ma12233824

**Published:** 2019-11-21

**Authors:** Ajit Pal Singh, Fei Yang, Rob Torrens, Brian Gabbitas

**Affiliations:** Waikato Centre for Advance Materials and Manufacturing, School of Engineering, University of Waikato, Hamilton 3216, New Zealand; fyang@waikato.ac.nz (F.Y.); torrens@waikato.ac.nz (R.T.); briang@waikato.ac.nz (B.G.)

**Keywords:** blended powder, vacuum sintering, extrusion, heat treatment, microstructure, toughness, fracture, crack propagation behaviour

## Abstract

The mechanical properties of titanium and titanium alloys are very sensitive to processing, microstructure, and impurity levels. In this paper, a blended powder mixture of Ti-6Al-4V alloy was consolidated by powder compact extrusion that involved warm compaction, vacuum sintering, and hot extrusion. The as-processed material with an oxygen content of 0.34 wt.% was subjected to various annealing treatments. The impact toughness of heat-treated material was determined using Charpy V-notch impact testing at room temperature. An emphasis was placed on establishing a relationship among fracture behaviour, microstructure, and the resulting properties of tested material. From the results, it is apparent that the highest impact toughness value of 19.3 J was achieved after α/β annealing and is comparable with typical values given in the literature for wrought Ti-6Al-4V. In terms of fracture behaviour, it is quite apparent that the crack propagation behaviour of powder-produced material is rather complex compared with the limited amount of data reported for ingot counterparts.

## 1. Introduction

The microstructures in material produced by powder metallurgy (PM) tend to be more complex than ingot-derived material, with more refined features. Furthermore, heat treatment of such microstructures results in more diversity in terms of grain and α/β colony size and the morphology of both lamellar and grain boundary α. Hence, finding a possible relationship among heat treatment, resulting microstructure, and mechanical properties is rather important in powder-produced material. Additionally, if relatively inexpensive powders are being used, then titanium and titanium alloys produced by PM route have much higher impurity contents compared to ingot counterparts. High oxygen content in the titanium lattice increases the strength, Young’s modulus, fatigue strength, and hardness, but compromises the ductility, impact strength, and fracture toughness. Therefore, it is very important to determine the level of enhancement or balance of properties that can be obtained from various heat treatments. The theme of this paper is to evaluate the effect of various heat treatments on material produced by powder compact extrusion (PCE) processing. 

Before specifically focusing on the current study, it is important to cover some of the background on the effect of microstructure on mechanical properties. Therefore, a review of previous studies on the impact toughness of ingot- and powder-produced Ti-6Al-4V alloy is presented. The relationship among heat treatment, resulting microstructure, and mechanical properties for ingot/wrought titanium and titanium alloys is well understood, and this information is readily found in the literature, in particular for Ti-6Al-4V alloy [1,2,3]. A general conclusion from an extensive body of literature suggests that a duplex microstructure is good for providing creep strength without excessively compromising fatigue strength, while a fine equiaxed microstructure gives high tensile strength, good ductility, and a good resistance to fatigue crack initiation. Other mechanical properties such as impact toughness, fracture toughness, resistance to fatigue crack propagation, and creep strength are associated with Widmanstätten/basketweave/lamellar-type microstructures [1,2,3].

There are a number of studies in the literature that investigated the effect of post-processing heat treatments on impact toughness of Ti-6Al-4V alloy produced by conventional ingot/wrought or casting routes [1,4,5,6]. The work done by Meyer et al. suggests that heat treatment of mill-annealed Ti-6Al-4V alloy gives a range of impact toughness; however, furnace cooling from the β or α + β phase fields provides the best energy absorption to failure, with values in the range 42–46 J, using U-notch specimens [6]. It is reported that an increase in the sharpness of the notch considerably decreases impact toughness [7]. Therefore, utilisation of a U-shaped notch generally provides a larger impact energy value and, thus, extra care should be taken when comparing results with other studies. 

Reda et al.’s work on the heat treatment of as-cast Ti-6Al-4V alloy reported significantly inferior impact strengths than those given in other studies on wrought counter parts [4,5]. A typical impact toughness of as-cast material in their work was around 11 J with various α + β heat treatments giving only a limited enhancement in toughness. The work done by Kwon et al. on heat treatment of rolled plate emphasised that annealing in the α + β phase field followed by ageing is more effective in improving impact absorbed energy (as high as 35 J) compared to material which underwent β annealing plus ageing, where an impact energy around 24 J was attained [8]. Similarly, a study by Buirette et at. on β-annealed and α/β-annealed rolled plate suggests that a lamellar microstructure gives a superior average fracture energy (19 J) to an equiaxed microstructure (13 J) [9]. Other than these studies, further impact toughness data published for heat-treated ingot/wrought/investment cast Ti-6Al-4V alloy typical vary between 17 and 46 J [1,10].

There are very little data for impact toughness of heat-treated Ti-6Al-4V alloy produced by a PM route, yet good impact toughness may be a requirement for many applications. Guo et al. reported that an impact toughness value of about 500 kJ/m^2^ (50 J/cm^2^ or 40 J) is achievable by heat-treating a powder compact originally produced by hot isostatic pressing pre-alloyed gas atomised powder with an oxygen level of 0.12 wt.% [11]. In this study, a heat treatment consisting of an annealing temperature of 930 °C, an isothermal hold of one hour, and subsequent air-cooling was employed prior to ageing. Additionally, U-notch Charpy impact specimens were used, which generally provide much higher impact energy compared to V-notch Charpy specimens. Another study by Yasa et al., which investigated the effect of heat treatment on selective laser-melted material, reported an impact energy of about 10.1 J after annealing for two hours at 735 °C [12]. There are few other studies that investigated the effect of microstructure on impact toughness. Among these, microstructures were altered using different processing conditions or processing routes, rather than by applying various post-processing heat treatments [13,14].

Another important aspect that lacks significant understanding is crack propagation behaviour of titanium and titanium alloys during impact toughness testing. There are several studies that investigated the fracture surfaces of Ti-6Al-4V alloy after impact testing [1,4,5,6,8,9]. However, there were only a limited number of studies that specifically investigated the crack propagation mechanism during Charpy impact toughness testing on titanium and titanium alloys. Recently, Buirette et al. made some effort to explore fracture and crack propagation behaviour of highly textured rolled plates of ingot Ti-6Al-4V alloy with an α equiaxed microstructure and a very coarse α lamellar microstructure (grain size in the range 920 ± 320 µm) [9]. No other similar studies for titanium and titanium alloys produced by a PM route were reported, despite the fact that PM-produced material has a more refined microstructure with some residual porosity or other processing-related defects, which result in significant differences in the fracture/crack propagation mechanism.

In the current study, four different heat treatments are employed to gain a better understanding of the Charpy impact behaviour of heat-treated Ti-6Al-4V alloy produced from a blended powder mixture with oxygen levels in the range of 0.33–0.35 wt.%. In addition, possible relationships between the microstructure and fracture behaviour of impact test specimens through an investigation of crack path and an in-depth examination of fracture surfaces are explored.

## 2. Material and Methods

In this study, titanium alloy rods with a nominal composition of Ti-6Al-4V were produced following a standard blended elemental (BE) processing sequence of blending, warm pressing, and vacuum sintering prior to hot extrusion. Information regarding the starting powders and processing parameters used in this study can be found in Table 1. The particle size distribution of starting powders was obtained using a Malvern Mastersizer 2000 analyser (Malvern Instruments Ltd., Worcestershire, UK).

Elemental hydride–dehydride (HDH) Ti powder was blended with 60Al-40V master alloy powder using a roller mill at a speed of 200 rpm for 24 hours in an air-tight container. The blended powder mixture was uniaxially warm pressed at 220 °C in a 56-mm-diameter H14 heat-treated steel die under a pressure of 313 MPa with 8 min of hold time to achieve 80% or above relative density for each of the powder compacts. The inner surface of this die and plunger was coated with colloidal graphite lubricant to reduce die wall friction. Prior to extrusion, these green compacts were sintered to 1325 °C with a heating rate of 10 °C/min using a vacuum furnace. The isothermal hold time was selected to be two hours, followed by natural cooling to room temperature. A vacuum in the range of 1–3 × 10^−2^ Pa was maintained inside the furnace during heating and isothermal holding. For powder compact extrusion, the vacuum-sintered compacts were heated again to temperatures in the range of 1150~1200 °C using an induction coil in air. The hot compact was shifted to a cylindrical extrusion chamber which, along with the die, was already at 420 °C. A 300-ton hydraulic press was used to produce rods with a diameter of 20 mm and extrusion ratio of 9:1.

**Figure 1:**
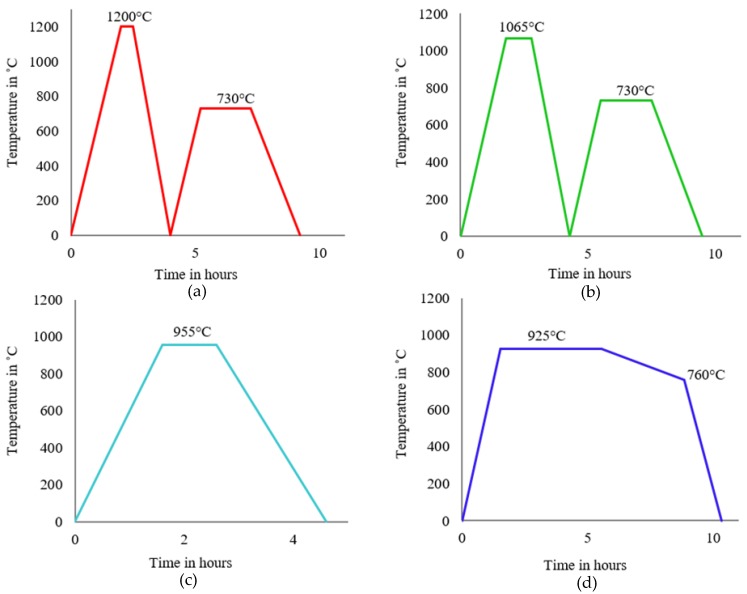
Schematic showing time-temperature cycles for annealing treatments (**a**) 1200 °C/30 min, FC, 730 °C/2 h; (**b**) 1065 °C/1 h, FC, 730 °C/2 h; (**c**) 955 °C/1 h, FC; (**d**) 925 °C/4 h, CFC@50 °C/h to 760 °C, FC. Note: FC—furnace cooling and CFC—controlled furnace cooling.

Extruded rods were produced as described in Table 1 and were then subjected to different heat treatments (see Figure 1). The annealing heat treatments were carried out in the same vacuum furnace used for sintering to avoid additional oxygen pickup. The functionality of this particular furnace does not allow for the removal of rods at high temperature; therefore, natural furnace cooling (FC) was used rather than air-cooling. For all annealing treatments, variations such as annealing temperature, isothermal hold time, ageing temperature, and cooling rate were incorporated. A heating rate of 10 °C/min was used for every heat treatment, followed by an isothermal hold. Cooling to 200 °C from any temperature above the β transus was reached in two hours once the furnace was turned off. The thermal cycle used for the four annealing treatments can be seen in Figure 1. The first treatment involved a high β anneal, followed by two hours of ageing in the α/β region. A more standard β annealing temperature (a few degrees higher than the β transus temperature) was used in the second treatment, followed by the same ageing scheme as in the previous case. The third case involved FC from the α/β region without any ageing step. Similarly, the final treatment was also α/β annealing, where there was well-controlled cooling from 925 to 760 °C, followed by FC.

The chemical compositions, impurity content, and microstructures of as-extruded and heat-treated materials were determined using X-ray fluorescence (Spectro Xepos spectrometer, SPECTRO Analytical Instruments, Kleve, Germany), an inert gas fusion method (ASTM E1409-08), and optical metallography (Olympus BX60, Olympus Optical Co Ltd, Tokyo, Japan), respectively. The ground and polished samples for optical metallography were etched in a modified Kroll’s reagent consisting of 2 vol.% HF, 4 vol.% HNO_3_, and 94 vol.% H_2_O. The heat-treated extruded rods were machined to obtain three impact toughness specimens with dimensions of 10 mm × 10 mm × 55 mm and a 2-mm-deep V-notch with an approximate tip radius of 0.25 mm, which was introduced using an electro discharge machine (EDM). Impact testing was performed at room temperature using an Avery-6703 impact tester (Avery Denison, United Kingdom) with a maximum energy rating of 300 J and an impact velocity of 5 m/s.

The fracture surfaces and crack propagation behaviour of heat-treated impact-tested specimens were examined using optical and scanning electron microscopy. A broken half of a fractured impact specimen was examined using a Carl Zeiss SEM, and multiple fractographs were captured at different magnifications to identify the mode of fracture and other key fracture features. Similarly, the relationship between crack propagation path and microstructure was explored using the other broken half of an impact test-piece (one from each heat-treated condition) which was sectioned perpendicular (longitudinal direction) to the crack propagation direction. In order to capture optical images of the crack path, a standard procedure of epoxy mounting, grinding, polishing, and etching was employed.

## 3. Results

The chemical composition and oxygen content of the material after various heat treatments are shown in Table 2 along with as-processed material (VS-E3’). The actual chemical composition of heat-treated Ti-6Al-4V rods was very consistent, and the concentration of Al and V was within the ranges 5.5–6.75 wt.% and 3.5–4.5 wt.%, as stated in the ASM standard for Ti-6Al-4V alloy [15]. 

The oxygen content of material produced in this study varied between 0.33 and 0.35 wt.%. A comparison of the oxygen contents achieved in this work with the specification required in the ASTM-B988 standard for powder-produced structural components suggests that the level of oxygen impurity attained here was marginally higher (0.03–0.05 wt.%) [16]. However, the impurity oxygen level here is much higher than that for commercially available grade five ingot Ti-6Al-4V (oxygen content ≤0.2 wt.%).

### 3.1. Microstructure

**Figure 2:**
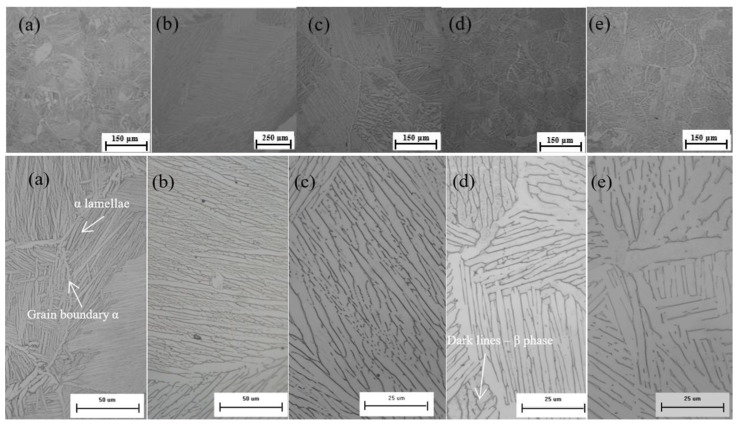
Optical micrograph of powder compact extrusion (PCE)-produced Ti-6Al-4V alloy: (**a**) as-extruded: fine lamellar microstructure; (**b**) 1200 °C/30 min, FC, 730 °C/2 h: coarse lamellar microstructure with a complex structure of α lamellas; (**c**) 1065 °C/1 h, FC, 730 °C/2 h: basketweave structure with partially broken up β phase; (**d**) 955 °C/1 h, FC: fine lamellar microstructure with complex grain and colony structure; (**e**) 925 °C/4 h, CFC@50 °C/h to 760 °C, FC: α laths, interfaces, and the grain boundary layer morphology. Note: the bottom row of images shows the same features at high magnification.

The microstructure of as-extruded material had a typical Widmanstätten/lamellar appearance which formed as a result of diffusion-controlled growth of the α phase as the material air-cooled from the β phase after exiting the extrusion die (see Figure 2a). Annealing above the β transus followed by ageing at 730 °C for two hours led to the formation of a coarse lamellar-type microstructure. Furnace cooling from a relatively high temperature (1200 °C) resulted in larger α + β colonies containing very coarse α lamellas, as shown in Figure 2b. In general, there were fewer colonies per grain, with some grains having a more complex colony morphology. The amount of retained β phase also varied from grain to grain. Most of the grains were well defined by a fine grain boundary α with uniform thickness. 

The standard β annealing treatment (1065 °C/1 h, FC, 730 °C/2 h) gave a microstructure containing a small, but very complex colony structure within larger grains. It is apparent that transformation from β to α led to a typical basketweave structure in most areas; however, there were a few areas that contained significant thick α plates separated by fine discontinuous dark lines corresponding to retained β, as shown in Figure 2c. This type of variation significantly affected the volume fraction of each phase along with the general morphology present within the same grain or colony. Overall, the grain size after this heat treatment was significantly larger (in the mm range) compared to material after a non-standard high β anneal and ageing treatment (1200 °C/30 min, FC, 730 °C/2 h). The combination of a high annealing temperature and an isothermal hold time were thought to be the reasons for the fast grain growth kinetics, resulting in very large grains with a significant variation in colony, α lath, and lamellar interfaces. 

Furnace cooling from 955 °C produced a homogeneous lamellar microstructure containing small α/β colonies, with complex orientations (Figure 2d). This microstructure had a high β phase content as the lamella packages were well defined by continuous and uniformly thick darker lines. Other than the β phase, the grain boundary α phase was thicker compared with that observed in previous annealed and aged microstructures. There were a few colonies which had a lighter appearance, and which showed less clear features. An investigation of these areas at high magnification indicated that these features contained some retained β phase in the form of a very fine discontinuous line or dots. 

This annealing treatment gave a similar homogeneous microstructure to that described for the previous case, with a few new key features. This microstructure had an even more complex colony arrangement containing a partly broken up β phase between each lenticular lamellar plate as shown in Figure 2e. Overall, the α morphology at the β boundaries was similar, but there were a few areas containing complex triple joints along with discontinuous grain boundary layers. There was also less retained β compared with that shown in the previous microstructure, which was evident from the thinner interfacial β (Figure 2e). It is clear that very slow controlled cooling from 975 °C to 760 °C at a rate of 50 °C/h followed by standard furnace cooling was responsible for α coarsening and a reduction in the β phase fraction. 

### 3.2. Impact Properties

The room temperature impact toughness of heat-treated Ti-6Al-4V alloy rods is summarised in Table 3. Here, impact toughness values for individual Charpy impact specimens is reported along with the mean, standard deviation (SD), and standard error (SE) obtained from three replicate tests. The impact toughness of the heat-treated material varied between 13.7 and 19.3 J on average. It is very evident that impact toughness for each heat-treated material had a significant standard deviation/standard error and, due to this fact, the effect of individual heat treatments that fell under the same general category was indistinguishable. A comparison of impact energy values from each of the heat treatments suggests that the incorporation of post α + β annealing and β annealing and ageing treatments improved the average energy absorption to failure. The Charpy V-notch impact energy of as-extruded Ti-6Al-4V alloy (VS-E3’) fabricated from a blended powder mixture using PCE was 13.7 ± 0.3 J. Material after α/β annealing gave a mean impact toughness 40% higher than that for as-extruded material. Similarly, annealing treatments above the β transus followed by ageing at 730 °C gave Charpy impact strengths of 1.3–1.7 J (11–14%) higher than the base material. 

Overall, each heat treatment category could be ranked as follows: α + β annealing (19.3 J) > β annealing plus ageing (15.3–15.7 J) > as-extruded (13.7 J), based on their positive effect on mean impact toughness.

### 3.3. Fracture Behaviour of Charpy Impact Specimens

**Figure 3:**
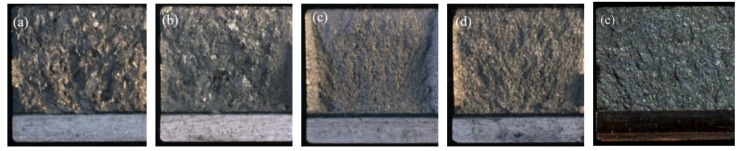
Fracture appearance of broken impact specimens: (**a**) 1200 °C/30 min, FC, 730 °C/2 h; (**b**) 1065 °C/1 h, FC, 730 °C/2 h; (**c**) 955 °C/1 h, FC; (**d**) 925 °C/4 h, CFC@50 °C/h to 760 °C, FC; (**e**) as-extruded.

The optical fractographs of impacted specimens for each heat treatment are shown in Figure 3. It is very apparent that fracture surfaces of both β-annealed and aged conditions were very rough with shiny facets and thin shear lips (Figure 3a,b). The general fracture appearance of α + β heat-treated material (955 °C/1 h, FC and 925 °C/4 h, CFC@50 °C/h to 760 °C, FC) were very similar. Here, the fracture surface was much smoother, with larger shear lips of more uniform thickness around the outer edges, as shown in Figure 3c,d. For as-extruded material (VS-E3’) shown in Figure 3e, the typical fracture surface was rather flat and featureless, and there were no shear lips around the outer edge, clearly suggesting greater brittleness compared to heat-treated materials.

#### 3.3.1. Crack Propagation Behaviour

**Figure 4:**
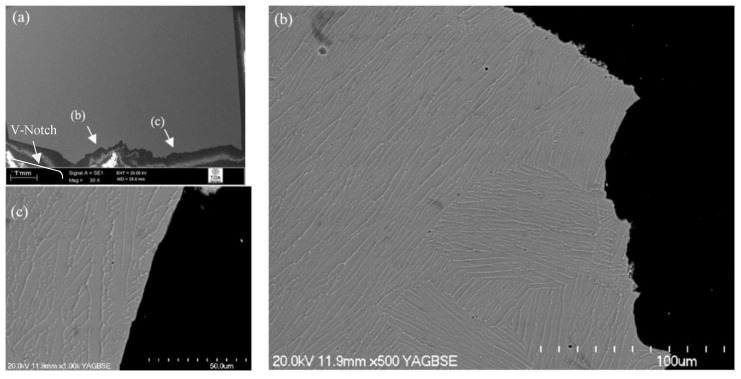
Crack propagation behaviour of 1200 °C/30 min, FC, 730 °C/2 h heat-treated material: (**a**) overall crack path; (**b**) crack path in first half; (**c**) crack propagation in second half (relative location of image (**b**) and (**c**) is marked on (**a**)).

The crack path profile for β-annealed and aged (1200 °C/30 min, FC, 730 °C/2 h) microstructure could be divided into two parts (Figure 4a). A rough and regularly deviating crack profile was observed in the first half of the fracture profile (Figure 4b), whereas, after reaching the middle of the sample, the crack propagation mechanism changed. From here onwards, the crack followed a straight line (clear sign of brittle fracture) (Figure 4c). Overall, the fracture mode was predominantly transgranular as there were no signs of cracking around the grain boundaries. At an early stage of crack propagation, the individual α + β colonies and thick α lamellas provided some resistance to crack advancement, along with the grain boundary α layer. In the remaining half of the fractured test-piece, the propagating crack ruptured all the microstructural features randomly, which suggests that an unstable fracture occurred. Overall, the large aspect ratios of the α lamellas and resulting colonies were more resistant to fracture. This is most likely because this microstructural feature increased the crack length and, therefore, the surface area or surface energy, giving a larger toughness.

**Figure 5:**
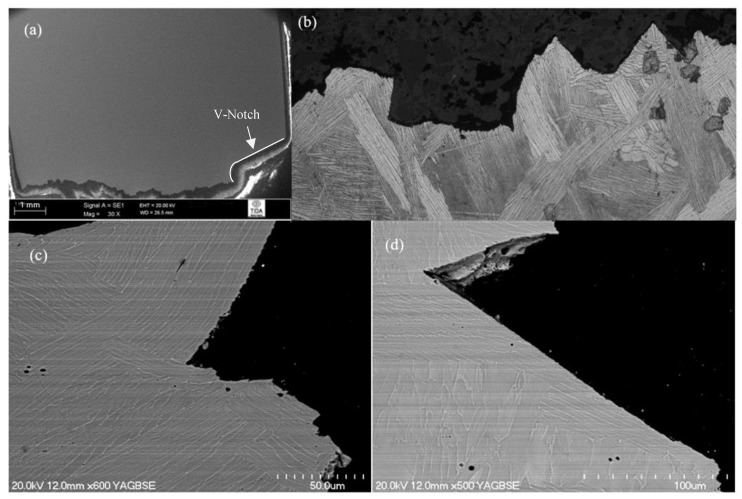
Crack propagation behaviour of 1065 °C/1 h, FC, 730 °C/2 h heat-treated material: (**a**) overall crack path; (**b**) transgranular fracture with relatively sharp crack deflection; (**c,d**) microstructural features causing a significant change to crack path.

The crack profile for material heat-treated 1065 °C/1 h, FC, 730 °C/2 h suggests a predominantly transgranular fracture mode in which the angle of crack front deviation was very sharp (as displayed in Figure 5a,b). The high-magnification SEM fractographs clearly indicate that the main sources of crack deflections were the entrance points to a new colony or, in some instances, a relatively thick individual α lamella arrested the crack, as shown in Figure 5c,d.

Generally, after entering each colony, cracks ruptured individual lamellas at a variety of angles to obtain the path of least resistance, and the thick grain boundary α layer played a negligible role in causing any change to the crack front. Overall, it can be concluded that relatively small colonies with large aspect ratio α lamellas (basketweave) did not further improve the crack propagation resistance compared with material heat-treated at 1200 °C/30 min, FC, 730 °C/2 h. Hence, the average impact toughness values for this heat treatment category were in close agreement.

**Figure 6:**
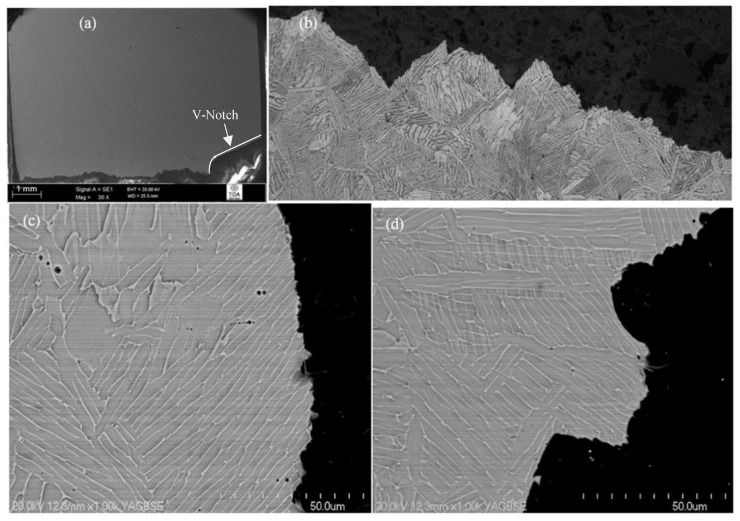
Crack propagation behaviour of 955 °C/1 h, FC heat-treated material: (**a**) overall crack path; (**b**) transgranular fracture; (**c,d**) individual α lamellar plates and retained β phase matrix providing sufficient hindering to crack growth.

The crack propagation behaviour of material furnace cooled from 955 °C (shown in Figure 6) was drastically different from that described in the previous two cases (β-annealed and aged). Overall, the crack path shown in Figure 6a highlights the fact that the amplitude of crack front deviation was relatively small, while changes in the crack path were much more frequent. This suggests that individual microstructural features caused significant resistance to crack propagation, while the crack spread through the interior of grains (as clearly observed in the optical fractographs in Figure 6b). Confirmation of this can be seen in the higher-magnification images shown in Figure 6c,d. From these images, it is clear that a crack was not only deflected by the differently orientated colonies, but each α lamellar plate and retained β phase matrix diverted the crack propagation paths to possibly retard crack propagation by blunting the crack tip. Additionally, the interaction of the crack with grain boundary α had a minor contribution to crack propagation resistance. In general, it can be concluded that a relatively thick retained β interface and individual α lamellar platelets in this microstructure were much tougher than those in β-annealed and aged material. Overall, each individual microstructural feature hindered the crack growth and, as a result, the best impact toughness values were obtained for this particular microstructure compared to the other heat treatments.

**Figure 7:**
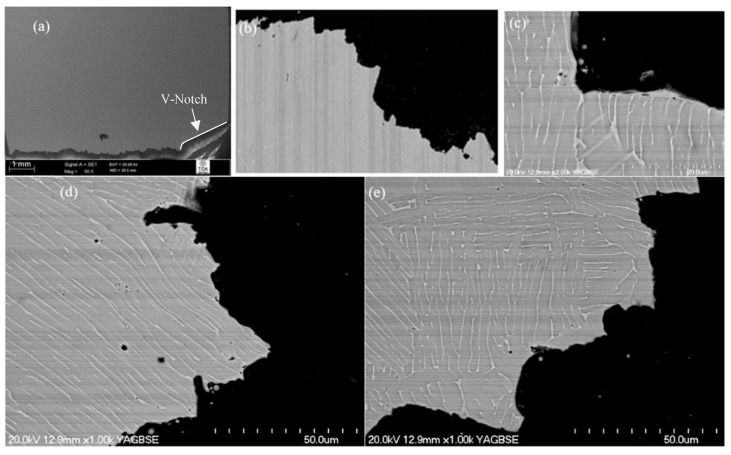
Crack propagation behaviour of 925 °C/4 h, CFC@50 °C/h to 760 °C, FC treated material: (**a**,**b**) overall crack path; (**c**) interaction with β phase; (**d**,**e**) different forms of α phase providing sufficient resistance to crack growth.

The general crack path in material annealed using 925 °C/4 h, CFC@50 °C/h to 760 °C, FC shown in Figure 7a,b was comparable to that described in the previous case (where the material was cooled from 955 °C). From high-magnification SEM fractographs (Figure 7c–e), it was clear that various forms of α phase, i.e., the thick α present in a more rounded form, individual lamellas, and those at colony interfaces, were responsible for changes in crack direction. The retained β phase also provided some resistance to crack propagation. However, in this case, there was only sufficient blunting at the crack tip when there was a head-on encounter by the crack with a non-uniform thickness β strip. An example of such behaviour is presented in Figure 7c, where the crack deviated towards the path of least resistance after interacting with the β phase. Most of the time, cracks ripped apart these β interface strips and, therefore, a local deflection of the crack after passing each individual lath was absent. Overall, transgranular fracture was the major failure mode as there was no indication of cracking around grain boundaries.

**Figure 8:**
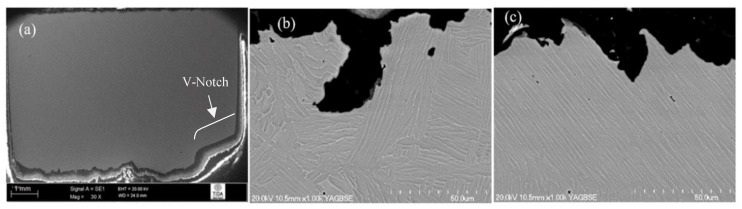
Crack propagation behaviour of as-extruded Ti-6Al-4V alloy: (**a**) overall crack path; (**b**) major deviation caused by colony interface; (**c**) limited resistance provided by retained β phase matrix.

For as-extruded material, an examination of the fracture surface of broken impact specimens (Figure 8) showed that the amplitude of the crack deflection was relatively lower than that for 925 °C/4 h, CFC@50 °C/h to 760 °C, FC annealed and 955 °C furnace-cooled samples. However, a phenomenon of frequent changes in crack path was still very active (see Figure 8b,c).

The zigzagging of the crack profile here was mainly due to the presence of small colonies and a relatively fine morphology of the individual α and β phases. The size of the shear lips was similar to that observed in β-annealed and aged (730 °C) samples, as well as in 955 °C quenched and aged samples. Overall, an energetically favourable fracture path in this sample was crack deflection, as the crack entered individual colonies and cut through the α lamellas and interfacial and grain boundary α layers at a range of angles.

#### 3.3.2. Fracture Surfaces 

**Figure 9:**
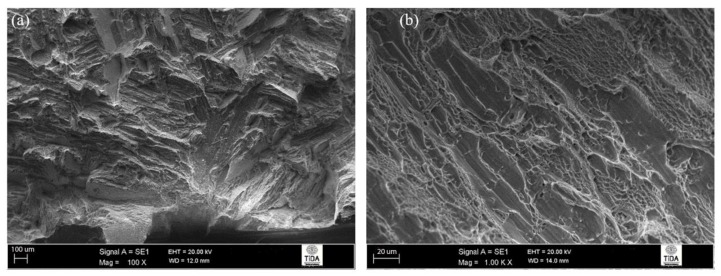
Fracture surface of 1200 °C/30 min, FC, 730 °C/2 h heat-treated Ti-6Al-4V alloy: (**a**) cleavage fracture; (**b**) ductile dimples.

After annealing at 1200 °C followed by ageing at 730 °C, the failure mode was mostly cleavage (see Figure 9a,b). There were just a few areas where crack propagation was trans-lamellar, and some ductile dimpling was evidence of a limited amount of ductile fracture. These observations correlate with the earlier crack path investigation studies, and the cleavage seen here derived from cracks travelling along crystallographic planes with a preferred orientation relating to colony interfaces.

**Figure 10:**
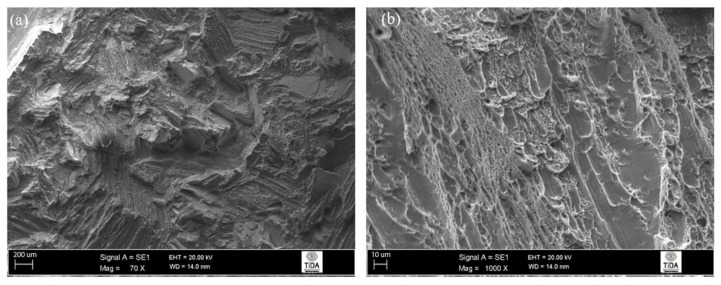
Fracture surface of 1065 °C/1 h, FC, 730 °C/2 h heat-treated Ti-6Al-4V alloy: (**a**) transgranular fracture marks; (**b**) signs of ductile fracture.

The features present in the fracture surface of standard β-annealed and subsequently aged samples (Figure 10a,b) included well-defined transgranular fracture features, some cleavage facets, and sufficient dimples to be comparable to those in high β-annealed and aged samples. The size of the spallings (areas where a crack travelled through the lamellas) were very similar to the colony size, and this confirmed the observations made regarding the path taken by the crack. 

**Figure 11:**
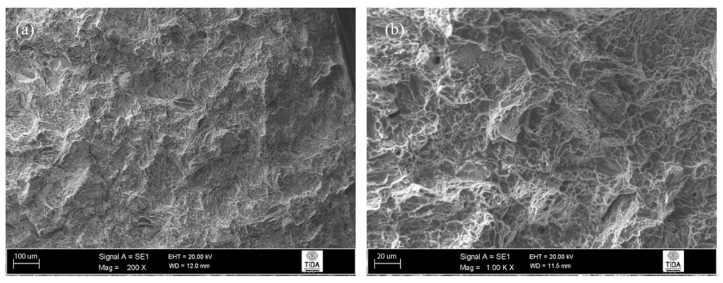
Fracture surface of 955 °C/1 h, FC heat-treated Ti-6Al-4V alloy: (**a**) general appearance; (**b**) evidence of ductile fracture.

Fractographs of fractured impact specimens given a 955 °C/1 h, FC heat treatment are shown in Figure 11, and these showed a typical ductile fracture that one would expect from a Ti-6Al-4V alloy. Here, the presence of many ductile dimples and ductile tearing ridges suggests that the predominant mode of failure was ductile. There are no signs of trans-lamellar fracture, which suggests that α platelets provided sufficient resistance to crack propagation. In other words, the individual α lamellas and β phase matrix were plastically deformed, giving a fracture surface that consisted mostly of ductile fracture features.

**Figure 12:**
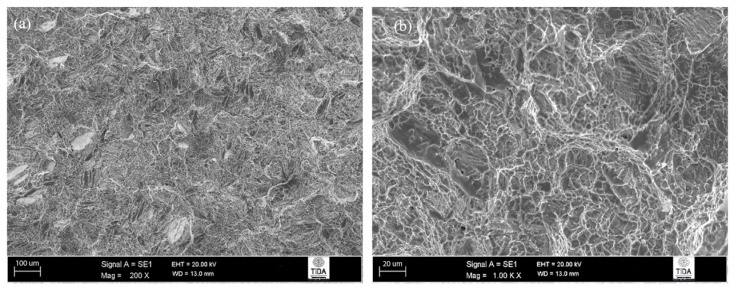
Fracture surface of 925 °C/4 h, CFC@50 °C/h to 760 °C, FC heat-treated Ti-6Al-4V alloy: (**a**) general appearance and the occasional presence of cleavage facets; (**b**) ductile dimpling.

The fracture surfaces of Ti-6Al-4V heat-treated using 925 °C/4 h, CFC@50 °C/h to 760 °C, FC looked rather ductile at first sight. However, the presence of well-built cleavage facets on the fracture surface cannot be ignored (as shown in Figure 12a). A comparison of this fracture surface with that for the previous case (where material was subjected to 955 °C/1 h, FC) suggests that, in this case, the cleavage fracture features were evidence of brittle behaviour along with smaller-sized dimples (compare Figure 11 and Figure 12). Despite this fact, the mean impact toughness values from each of these α + β annealing treatments was the same.

**Figure 13:**
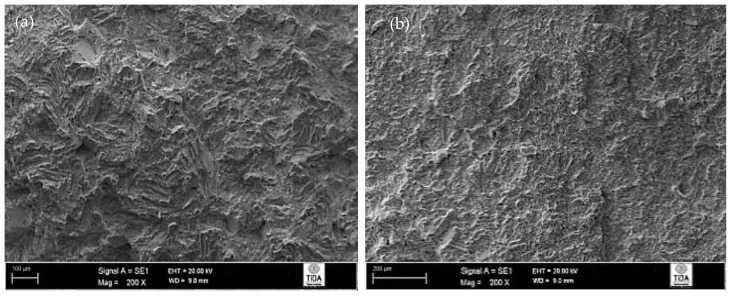
Typical fractographs of as-extruded Charpy impact specimens: (**a**) cleavage and transgranular fracture; (**b**) relatively flat surface with brittle fracture.

For as-extruded material, the fracture area containing ductile dimples or other signs of ductile fracture was significantly smaller than the area represented by cleavage and transgranular fracture in all surfaces investigated. Hence, it was very evident that brittle fracture was the predominant mode of failure here. Figure 13a,b show high-magnification fractographs that contain a significant number of cleavage facets along with large plane spallings (or transgranular features).

In general, the type of fracture features present in both α + β-annealed materials emphasises the point that the energy required to fracture an α colony (or α platelets) is greater than that required to circumvent them. Therefore, a much more frequent change in crack path was observed because cracks had to deviate past the colonies or around α platelets, causing significant plastic deformation that resulted in ductile failure. Overall, it is very clear that there was more pronounced ductile dimpling here compared with all the other samples investigated.

## 4. Discussion

There are many potential applications of titanium and titanium alloys requiring lower but reproducible levels of mechanical properties. For this reason, Peter et al. suggested that an industrial grade of titanium and titanium alloys should be developed, which can provide adequate levels of properties to fulfil the obligation of less demanding components rather than manufacturing of these parts from non-feasible aerospace-quality material [17]. Based on this principle, the present study explores the processing of Ti-6Al-4V alloy prepared from starting powders with relatively high oxygen contents. To further enhance the economics, a blended elemental powder approach was employed to produce Ti-6Al-4V alloy powder, which was then consolidated using processing steps such as warm pressing, vacuum sintering, and hot extrusion in air. 

The results given in this paper show that microstructural control of Ti-6Al-4V alloy, produced from a relatively high-oxygen-containing blended powder mixture via PCE processing, is necessary for achieving levels of impact toughness which are sufficiently high enough to fulfil the requirements of typical applications. It can be concluded that heat treatments such as furnace cooling from 955 °C (without any ageing) and 925 °C/4 h, CFC@50 °C/h to 760 °C, FC are capable of achieving an optimum microstructure that delivers the best impact toughness. Also, the level of properties obtained after such heat treatments closely matches the expected values of ingot or wrought counterparts, even though the oxygen contents of powder-produced material in this study are still 0.13–0.15 wt.% higher than those in typical grade five Ti-6Al-4V. 

### 4.1. Microstructure Comparison

The microstructure of as-extruded, β-annealed plus aged, and α + β-annealed material was generally characterised as a typical lamellar/Widmanstätten /basketweave-type structure. From each individual optical micrograph, it was clear that a significant difference in microstructural features, such as thickness, length and aspect ratio of α laths, α/β colony size, and thickness of grain boundary α does exist. Generally, the morphology of this microstructure attained after 1200 °C/30 min, FC, 730 °C/2 h was consistent with that reported in previous studies, where extra-low interstitial Ti-6Al-4V alloy was heat-treated under identical conditions [18]. However, the size of individual features in the microstructure described here were different, mainly due to the prior thermal history of the material and the different oxygen content. Furnace cooling from the β phase field gives a very coarse lamellar structure [1,3,19,20]. Here, for 1065 °C/1 h, FC, 730 °C/2 h treatment, the time of ageing at a relatively high temperature was responsible for a reduction in lamellar length and the formation of a complex colony arrangement within each grain, where a limited number of small aspect ratio laths were present. There were a few studies in which similar heat treatments were used for ingot/wrought or PM-produced material [6]. In these cases, the reported microstructures were significantly different from those observed in this work, where the material had a higher oxygen content and a relatively finer starting lamellar microstructure (or microstructure of as-processed material) [6]. 

A comparison of the phase morphology from 955 °C/1 h, FC heat treatment with the microstructure produced by prior thermomechanical processing suggests that the original α lamella length underwent minimal change, and the slow furnace cooling from the α + β region increased the α lamellar thickness. Meyar et al. carried out the same heat treatment on mill-annealed Ti-6Al-4V alloy, and their results were significantly different [1,3,6]. In their work, a duplex microstructure was obtained rather than a coarse lamellar structure as found in this work. The variation in starting microstructure is probably the main reason for such diverse results. Jia et al. used 925 °C/4 h, CFC@50 °C/h to 760 °C, FC heat treatment on as-forged HDH Ti-6Al-4V alloy, and their results were significantly different [21]. In their work, a fully equiaxed microstructure was obtained rather than a coarse lamellar structure. This suggests that the starting microstructure and prior thermal history, along with a higher level of impurity, have a significant effect on the final heat-treated microstructure. In other words, Ti-6Al-4V alloy prepared using the powder consolidation route described here has a very different as-processed microstructure compared to powder-forged parts. Therefore, a heat treatment established for powder-forged Ti-6Al-4V alloy cannot always be directly applied to other powder-produced material to obtain the same microstructural features.

### 4.2. Comparison of Impact Properties with Literature

To get a general idea of the level of impact toughness found in this study, a summary of previously published data for heat-treated Ti-6Al-4V alloy produced via various processing routes is considered in Table 4. It is important to realise that there are no reports of previous studies giving impact data for Ti-6Al-4V alloy which underwent severe powder particle deformation, such as that from PCE, and with the same impurity levels as the material in this work. Therefore, in this section, only general comments are made to put the current findings into context with a broad spectrum of impact energy values available for standard grade five heat-treated Ti-6Al-4V alloy. From Table 4, it is very apparent that the best impact toughness (19.33 J), obtained by furnace-cooling material from 955 °C or by a 925 °C/4 h, CFC@50 °C/h to 760 °C, FC treatment in this work, is 14% higher than the minimum value reported for typical grade five Ti-6Al-4V alloy [15].

Similarly, impact energy values obtained after each heat treatment in this study are better than values reported by Reda et al. on as-cast Ti-6Al-4V alloy and Yasa et al. who worked on heat treated selective laser-melted material [4,5,12]. However, the other impact toughness values presented in Table 4 are significantly higher than those obtained in this work, mainly because of the variation in oxygen content, processing route, or heat treatment conditions, along with inconsistency in notch shape and dimensions of test specimens [1,6,8,10,22].

**Figure 14:**
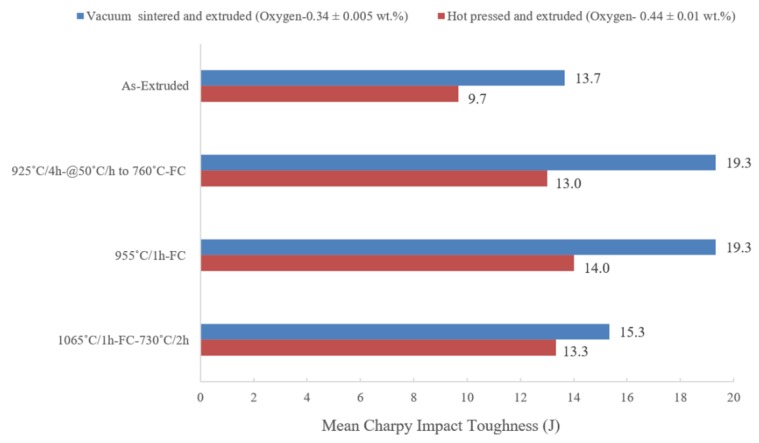
A comparison of mean Charpy impact toughness of heat treated Ti-6Al-4V alloy prepared via two different processing routes with different oxygen contents [23].

In addition to the comparisons with a broad spectrum of previously published data on ingot Ti-6Al-4V alloy (with oxygen 0.2 wt.%), it is also important to correlate the results from this work with research carried out previously by the author on hot-pressed and extruded material with an oxygen content of 0.44 wt.% [23]. In this work, a high-oxygen blended powder mixture was consolidated by hot-pressing a green compact prior to extrusion. Again, it is important to realise that the oxygen content and pre-consolidation technique (vacuum sintering or hot pressing) is different for each study. Therefore, a direct comparison is still not possible. A general observation made from Figure 14 suggests that the overall response of the material with respect to each heat treatment is similar. In both studies, furnace cooling from 955 °C appears to be the most effective heat treatment, as the highest impact energy absorptions to failure of 19.33 J and 14 J, respectively, were obtained for vacuum-sintered and extruded (oxygen 0.34 ± 0.005 wt.%) and hot-pressed and extruded (oxygen 0.44 ± 0.01 wt.%) material.

From Figure 14, it is also apparent that the heat-treated material described in this paper, with 0.1 wt.% lower oxygen, has (on average) a 2–6.3 J higher impact energy compared to hot-pressed and extruded material, depending on the respective microstructure/heat treatment. From the literature, it is clear that the presence of oxygen is favourable for some properties, whereas it is detrimental for others [1,2,3]. Generally, high oxygen content in the titanium lattice increases the yield strength, ultimate tensile strength, Young’s modulus, fatigue strength, and hardness, but compromises the ductility, impact strength, and fracture toughness. The main reason for a high dependency of the mechanical properties of titanium and titanium alloys on oxygen content can be explained by the interaction of oxygen atoms with the hydrostatic fields of both edge and screw dislocations and the alteration it brings to twinning and prismatic slip.

On the whole, it is clear that the levels of impact toughness given in this work match typical values stated for standard ingot grade five Ti-6Al-4V alloy with an oxygen content of 0.2 wt.%. The impact toughness is also significantly better than that for material processed by hot pressing and extrusion [23]. Hence, Ti-6Al-4V alloy with a high oxygen content ~0.34 wt.% produced in this study by PCE processing can be used for industrial applications after α + β annealing to satisfy the performance requirement of more critical applications. This offers an opportunity to expand the application of Ti-6Al-4V parts with high oxygen content, in addition to their utilisation in “fit-for-purpose” components.

### 4.3. Comments about Complex Fracture Behaviour in PM materials

The result attained in this study suggests that the global fracture morphology of each impacted specimen correlates well with respective microstructural features and resulting properties. In terms of crack propagation behaviour, each microstructure gave a completely different fracture path of least resistance. The general crack path in specimens with a lamellar type microstructure is rather tortuous, indicating that when a crack meets α platelets of suitable thickness or an interface between colonies, it has to follow the more energetically favourable pathway by either deflecting past the α lamellar platelets/colonies or by cutting through them. Similarly, from fracture surfaces observation, it was quite evident that most impact samples showed a mixed fracture mode, and there was confirmation of this in the form of well-defined cleavage facets, inter-granular fracture marks, and ductile dimples or ductile tearing in each case. Generally, it can be concluded that a fully lamellar or Widmanstätten-type microstructure of PM materials increases the resistance to crack propagation and results in a significant improvement in impact energy values.

The number of studies investigating crack propagation mechanisms in titanium and titanium alloys during a Charpy impact toughness test is very limited. Therefore, an in-depth discussion/comparison of the crack propagation behaviour of PM-produced Ti-6Al-4V with an ingot-produced and heat-treated alloy is not possible, except for a comparison with the limited work reported by Buirette et al. [9]. In their study, highly textured rolled plates of ingot Ti-6Al-4V alloy with a very coarse α lamellar microstructure (grain size in the range 920 ± 320 µm) were studied for crack propagation mechanisms. The general appearance of the crack path shows some similarity with the crack profile observed for heat-treated Ti-6Al-4V alloy described in this paper [9]. However, it is important to note that the more refined microstructural features in powder-produced Ti-6Al-4V alloy give complex fracture or crack propagation mechanisms leading to more frequent crack deviation possibilities. The frequent change in crack path is a beneficial phenomenon, because it simply highlights the fact that a larger amount of work is done in order to break the specimen into two halves. In other words, a significant resistance to crack propagation results in overall improvement in toughness of the respective material. 

Additionally, when considering the crack propagation mechanism, it was observed that most of the crack branching, deflection, crack-tip blunting, and microcrack formation in the current study was obtained from colony arrangement/orientation, α platelets, and retained β phase morphology. The role of prior β grain boundary in causing significant deviation in the crack path was negligible. This is very different to what was reported by Buirette et al. for ingot Ti-6Al-4V material; according to them, cracks were significantly arrested by a prior β grain boundary, in addition to colony interface or α laths [9]. This illustrates that there is some fundamental difference in crack propagation mechanism when it comes to the complex refined lamellar morphology of powder-produced materials compared to conventional ingot/wrought counterparts. 

## 5. Conclusions 

This research proves that it is possible to enhance the mechanical properties of Ti-6Al-4V alloy, produced by consolidating a blended powder mixture through a combination of vacuum sintering and extrusion, despite the presence of relatively high impurity oxygen contents (~0.34 wt.%). It can be concluded that annealing in high α + β phase followed by controlled cooling is the most effective heat treatment capable of achieving an optimum lamellar/Widmanstätten-type microstructure that delivers superior impact toughness levels equivalent to those found in ingot/wrought material. In terms of fracture, it can be stated that the crack deflection mechanism of the powder-produced material during impact toughness testing is rather complex, and it is different compared to the limited work reported for Ti-6Al-4V alloy produced by ingot metallurgy. Overall, Ti-6Al-4V alloy produced in this study meets the performance requirement for a variety of industrial applications, from “fit-for-purpose” parts to more critical components.

## Figures and Tables

**Table 1:** Characteristics of the starting powders and processing conditions used to produce Ti-6Al-4V alloy rods by powder compact extrusion (PCE). HDH—hydride–dihydride; MA—master alloy.

**Starting Powders**					
**Powders**	**Oxygen Content (wt.%)**	**Volume Fraction Below Particular (μm)**			**Particle Size**
		**d(0.1)**	**d(0.5)**	**d(0.9)**	
HDH Ti	~0.25	27.043	51.252	89.485	−200 mesh
MA (60Al-40V)	~0.61	28.384	53.460	91.167	−250 mesh
**Processing**					
**Process**	**Equipment**	**Description**			
Blending/roller mixing	Horizontal rollers	450 g of HDH Ti + 50 g of MA (60Al-40V) were mixed			
Compaction	100-ton hydraulic press	Warm compaction was performed at 220 °C, using 313 MPa of uniaxial pressure with 8 min of hold time			
Sintering	Vacuum furnace	All samples were sintered together at 1325 °C with a 10 °C/min heating rate and 120 min of hold time			
Induction heating	Induction furnace	Compacts were heated to 1150–1200 °C prior to extrusion			
Extrusion	300-ton horizontal hydraulic press	An extrusion speed of 122 mm/s was used along with a 9:1 extrusion ratio			

**Table 2:** A summary of chemical composition and oxygen contents of the extruded Ti-6Al-4V rods after various heat treatments.

Heat Treatment Description	Abbreviation (Treatment Temperature/Hold Time, Cooling Type, Ageing Temperature/Hold Time)	Composition (wt.%)			
		Ti	Al	V	O
-	As-extruded (VS-E3’)	90.5	5.8	3.6	0.34
β annealing + ageing treatment	1200 °C/30 min, FC, 730 °C/2 h	89.6	6.4	3.6	0.33
	1065 °C/1 h, FC, 730 °C/2 h	89.9	6.5	3.5	0.34
α + β annealing	955 °C/1 h, FC	89.9	6.3	3.6	0.35
	925 °C/4 h, CFC@50 °C/h to 760 °C, FC	90.3	6.0	3.6	0.33

Note: FC—furnace cooling, VS-E3’—as-extruded rod, CFC—controlled furnace cooling.

**Table 3:** Impact toughness of heat-treated Ti-6Al-4V alloy rods produced by powder compact extrusion (PCE).

Heat Treatment Description	Abbreviation (Treatment Temperature/Hold Time, Cooling Type, Ageing Temperature/Hold Time)	Impact Toughness (J)					
		S1*	S2	S3	Mean	SD^^^	SE^+^
-	As-extruded (VS-E3’)	13	14	14	13.67	0.58	0.33
β annealing + ageing treatment	1200 °C/30 min, FC, 730 °C/2 h	16	16	15	15.67	0.58	0.33
	1065 °C/1 h, FC, 730 °C/2 h	17	14	15	15.33	1.53	0.88
α + β annealing	955 °C/1 h, FC	20	20	18	19.33	1.15	0.67
	925 °C/4 h, CFC@50 °C/h to 760 °C, FC	20	19	19	19.33	0.58	0.33

Note: FC—furnace cooling, VS-E3’—as-extruded, CFC—controlled furnace cooling, S1*—impact specimen one, (SD^^^)—standard deviation, (SE^+^)—standard error.

**Table 4:** A summary of previously published impact toughness data for heat-treated Ti-6Al-4V alloy produced via various processing routes (including ingot or powder metallurgy).

Starting Material	Heat Treatment Category	Microstructure	Impact Toughness (J)	Comment	Reference
PCE	β-annealed and aged	Lamellar or basketweave	15.33–15.67	O ~0.34 ± 0.005 wt.%	Current work
	α + β-annealed	Lamellar	19.33		
	Quenched and aged	Martensitic or mixture of primary α, secondary α and β phase	13.67		[24]
TPM^+^	As-processed	Lamellar, Widmanstätten	9.7–14.3	O < 0.32–0.43 wt.%	[25,26]
Ingot	-	-	17	O < 0.20 wt.%	[15]
Ingot	Mill-annealed	-	20–27	O < 0.20 wt.%	[10]
Ingot (ELI)		-	20–40	O < 0.13 wt.%	[10]
HIPed investment cast	α + β-annealed plus ageing	-	28.2–30.6	O between 0.16–0.19 wt.%	[1,8]
	Β-annealed plus ageing	-	26–28.7		
As-casted	Quenching	Mixture of acicular αʹ, β structures and primary α	5–10	O ≤ 0.20 wt.%	[4,5]
	Duplex	Widmanstätten	8–12		
Rolled plate	α + β-annealed	Biomodal	35	O ≤ 0.186 wt.%	[7]
	β-annealed	Lamellar	24		
Rolled plate	α + β-annealed	Equiaxed	13	non-standard v-notch sample, O ≤ 0.20 wt.%	[12]
	β-annealed	Coarse lamellar	19		
Mill-annealed *	Quenched and aged	Martensitic microstructure or primary α phase plus quenched and aged	16–28	Impact Specimens with U notch *, Oxygen 0.18 wt.%	[6]
	Annealed	Coarse-grained lamellar or Duplex microstructure	42–46		
	α + β-annealed + aged	Biomodal	50 J/cm^2^ (40 J)		
	Annealing 735 °C for two hours	-	10.1		
HIPed PREP*	Annealing 735 °C for two hours	-	10.1	U-notch * Charpy specimens, oxygen < 0.12 wt.%	[22]
Selective laser melted	Annealing 735 °C for two hours	-	10.1	-	[12]
Electron beam melting	HIPed	-	3.78–57.73	O between 0.11–0.46 wt.%	[27]

Note: -: no information available, TPM+: thermomechanical powder processing, *: utilisation of U-notch Charpy impact specimen generally predicts much high-energy value compared to sharp V- notch.
